# Effect of Tantalum Pentoxide Addition on the Radiopacity Performance of Bi_2_O_3_/Ta_2_O_5_ Composite Powders Prepared by Mechanical Milling

**DOI:** 10.3390/ma14237447

**Published:** 2021-12-04

**Authors:** Hsiu-Na Lin, Chung-Kwei Lin, Pei-Jung Chang, Wei-Min Chang, Alex Fang, Chin-Yi Chen, Chia-Chun Yu, Pee-Yew Lee

**Affiliations:** 1 Research Center of Digital Oral Science and Technology, College of Oral Medicine, Taipei Medical University, Taipei 110, Taiwan; tiffanylin1214@gmail.com (H.-N.L.); chungkwei@tmu.edu.tw (C.-K.L.); peronchang@tmu.edu.tw (P.-J.C.); weiminchang@tmu.edu.tw (W.-M.C.); chencyi@fcu.edu.tw (C.-Y.C.); 2 Department of Dentistry, Chang Gung Memorial Hospital, Taipei 110, Taiwan; 3 School of Dental Technology, College of Oral Medicine, Taipei Medical University, Taipei 110, Taiwan; 4 Graduate Institute of Manufacturing Technology, National Taipei University of Technology, Taipei 111, Taiwan; 5 School of Oral Hygiene, College of Oral Medicine, Taipei Medical University, Taipei 110, Taiwan; 6 Department of Engineering Technology and Industrial Distribution, Texas A&M University, College Station, TX 77843, USA; gpafang@tamu.edu; 7 Department of Materials Science and Engineering, Feng Chia University, Taichung 407, Taiwan; 8 Center of Dental Technology, Chang Gung Memorial Hospital, Linkou, Taoyuan 333, Taiwan; 9 Department of Optoelectronics and Materials Technology, National Taiwan Ocean University, Keelung 202, Taiwan

**Keywords:** bismuth oxide, tantalum pentoxide, high temperature metastable phase, mechanical milling, radiopacity, diametral tensile strength, setting time, biocompatibility

## Abstract

Among the various phases of bismuth oxide, the high temperature metastable face-centered cubic δ phase attracts great attention due to its unique properties. It can be used as an ionic conductor or an endodontic radiopacifying material. However, no reports concerning tantalum and bismuth binary oxide prepared by high energy ball milling and serving as a dental radiopacifier can be found. In the present study, Ta_2_O_5_-added Bi_2_O_3_ composite powders were mechanically milled to investigate the formation of these metastable phases. The as-milled powders were examined by X-ray diffraction and scanning electron microscopy to reveal the structural evolution. The as-milled composite powders then served as the radiopacifier within mineral trioxide aggregates (i.e., MTA). Radiopacity performance, diametral tensile strength, setting times, and biocompatibility of MTA-like cements solidified by deionized water, saline, or 10% calcium chloride solution were investigated. The experimental results showed that subsequent formation of high temperature metastable β-Bi_7.8_Ta_0.2_O_12.2_, δ-Bi_2_O_3_, and δ-Bi_3_TaO_7_ phases can be observed after mechanical milling of (Bi_2_O_3_)_95_(Ta_2_O_5_)_5_ or (Bi_2_O_3_)_80_(Ta_2_O_5_)_20_ powder mixtures. Compared to its pristine Bi_2_O_3_ counterpart with a radiopacity of 4.42 mmAl, long setting times (60 and 120 min for initial and final setting times) and 84% MG-63 cell viability, MTA-like cement prepared from (Bi_2_O_3_)_95_(Ta_2_O_5_)_5_ powder exhibited superior performance with a radiopacity of 5.92 mmAl (the highest in the present work), accelerated setting times (the initial and final setting time can be shortened to 25 and 40 min, respectively), and biocompatibility (94% cell viability).

## 1. Introduction

Ever since Benjamin first synthesized oxide dispersion strengthened superalloys by mechanical alloying (MA) [1], the high energy ball milling process presented in his work has been widely used to prepare materials that are difficult to synthesize by conventional melting and casting techniques [2,3,4]. Starting with a mixture of various elements or powders, mechanical alloying occurs gradually by repetitive mechanical mixing, cold welding, fracturing, and rewelding of the mixed powders during ball–powder collision events [2]. When using a ductile metallic powder mixture, a lamellar structure can be observed, refined continuously, and results in a homogeneous phase or phases. If brittle and ductile powders are used together, fractured brittle powder will be embedded within ductile materials, refined continuously, and mechanically alloyed or forming a homogeneous composite at the end of process [5,6], whereas brittle materials alone will crack into pieces, entangle with each other, and be progressively refined. Reaction (with the assistance of impact energy) between starting brittle powders is also observed. Alternative processes based on high energy ball milling include mechanical milling [7,8] and mechanochemical synthesis [9,10]. The former starts milling with a single brittle powder or mixture, whereas the latter involves chemical reaction during the process. MA and its alternative techniques have been used widely to prepare numerous metastable materials including amorphous materials, extended solid solutions, intermetallic compounds, and quasicrystals [3,4,5,6,7,11].

High temperature metastable phases such as bismuth oxide-based materials can be prepared by high energy ball milling processes. Bismuth oxide can be used either as an ionic conductor in solid oxide fuel cells [12,13,14] or as a radiopacifier within endodontic filling mineral trioxide aggregates (i.e., MTA) [15,16,17] and has attracted considerable research and development interest [18,19]. Bismuth oxide possesses four different phases (α-, β-, γ-, and δ) [20,21]. Among them, the high temperature face-centered cubic δ-phase (only stable at temperatures ranging from 729 to 825 °C) is one of the best oxide ionic conductors. Oxides with higher valence cations or smaller ionic radii are preferred additions to bismuth oxide to help it retain the desired δ-phase at room temperature. For instance, hafnia and zirconia have been added in order to prepare a high temperature δ-phase [22]. Niobium or tantalum pentoxide were used to synthesize Bi_3_NbO_7_ and Bi_3_TaO_7_, respectively. Ternary Bi_2_O_3_-Nb_2_O_5_-Ta_2_O_5_ and Bi_2_O_3_-TiO_2_-WO_3_ oxide systems have also been investigated, and formation of metastable Bi_3_Nb_1-x_Ta_x_O_7_ and Bi_6_Ti_5_WO_22_ phases can be achieved [23].

When bismuth oxide is used as the radiopacifier within MTA, its performance is mainly affected by the atomic number of the radiopacifier. The crystalline structure of bismuth oxide, however, may not be a major concern. Presently, bismuth oxide (Bi_2_O_3_) is used as the radiopacifier within commercially available ProRoot^®^, whereas zirconium oxide and tantalum pentoxide are alternative radiopacifiers in commercial products of Biodentine^®^ and BioAggregate^®^, respectively. Previous investigations concerning mechanical milling of a Bi_2_O_3_-ZrO_2_ powder mixture resulted in the formation of a δ-Bi_7.38_Zr_0.62_O_2.31_ phase. The effect of milling time, zirconia addition, and storage environment on the radiopacity performance of the as-milled bismuth/zirconium oxide composite powders were addressed [24]. In addition, bismuth oxide was also mechanically milled with tantalum and high temperature β-Bi_7.8_Ta_0.2_O_12.2_, and a δ-Bi_3_TaO_7_ phase formed as a result of the mechanochemical reaction between Bi_2_O_3_ and Ta [25]. The radiopacity of these bismuth/tantalum oxide powders, however, was not investigated. Tantalum pentoxide added to bismuth oxide prepared by high energy ball milling and serving as a dental radiopacifier has not been reported in the literature. In the present study, high energy ball milling will be used to process Bi_2_O_3_ and Ta_2_O_5_ powder mixtures. The structural evolution of the high temperature bismuth oxide phase formation during ball milling was examined by 5 and 20 wt.% tantalum pentoxide addition (the smallest and largest addition in the present work). The radiopacity performance and diametral tensile strength (DTS) of MTA-like cements were examined as a function of Ta_2_O_5_ additions (5, 7.5, 10, 15, and 20 wt.%) An optimized composition will be explored further by adding various solidified solutions. In addition to the radiopacity and DTS, setting times and selected biocompatibility were investigated.

## 2. Materials and Methods

The starting powders for the mechanical milling process were commercially available α-Bi_2_O_3_ (99.9%, STREM, Newburyport, MA, USA) and tantalum pentoxide (β-Ta_2_O_5_, 99.98%, Wako Pure Chemical Industries, Ltd., Osaka, Japan) powders. A 4 g mixture of Bi_2_O_3_ and Ta_2_O_5_ with desired compositions of (Bi_2_O_3_)_95_(Ta_2_O_5_)_5_ and (Bi_2_O_3_)_80_(Ta_2_O_5_)_20_ in weight percentage and 20 g of Cr steel balls (7 mm in diameter) were canned into an SKH 9 high speed steel vial (40 mm in diameter and 50 mm in height). A high energy ball milling process was performed with an SPEX 8000D shaker ball mill (Fisher Scientific, Ottawa, ON, Canada). All experiments were operated under ambient atmospheric conditions. The mechanical milling process was initially set at 3 h and can be extended to 10 h for the formation of the metastable face-centered cubic phase. Typically, for the first 30 min of milling, the process was on and off at intervals of 5 min. The interval was increased to 30 min thereafter to the end of milling. At various milling stages, a suitable amount of the as-milled powder was extracted for structural characterization by X-ray diffraction (XRD) and scanning electron microscopy (SEM). A PANalytical X’PERT PTO diffractometer (Malvern Panalytical Ltd., Malvern, Worcestershire, UK) was used to examine the as-milled powder using monochromatic Cu Kα radiation generated by a voltage of 40 kV and an anode current of 30 mA. The XRD patterns were further investigated by the Rietveld fitting method using XRD analysis software EVA (Version 4.1.1, Bruker-AXS Diffrac EVA, Bruker, WI, USA) to determine the phase percentages at various milling stages. A Hitachi S-4800 field emission scanning electron microscope (Hitachi, Tokyo, Japan) was used to observe the cross sections of as-milled powders.

Selected as-milled powders were used as radiopacifiers to prepare MTA-like cements by mixing 75 wt.% Portland cement, 20 wt.% radiopacifier, and 5 wt.% gypsum with a planetary ball mill (PM100, Retsch, Haan, Germany) for 10 min. The MTA-like cement was solidified by adding deionized water, sterile 0.9% saline solution (abbreviated as saline), or 10 wt.% calcium chloride solution according to a powder to solution ratio of 3:1. Before solidification, the paste was placed into acrylic molds (10 mm in diameter and 1 mm in thickness for radiopacity test), and set at 37 °C for 24 h to prepare the MTA-like cements. Six samples were prepared for each test condition. Each set of MTA-like cements (N = 6) and an aluminum step-wedge (2–16 mm at an increment of 2 mm) were examined simultaneously by a dental X-ray system (VX-65; Vatech Co, Yongin Si Gyeonggi-Do, South Korea) that operated at a voltage of 62 kV, a current density of 10 mA, an exposure time of 0.64 s at a focus-film distance of 30 cm, and recorded by a dental image plate (Imaging plate size 2; Dürr Dental, Bietigheim-Bissingen, Germany). The images were processed by an imaging processing software (Image J 1.52a, Wayne Rasband, National Institutes of Health, Bethesda, MD, USA) to determine the corresponding radiopacity of the MTA-like cements by matching and interpolating the gray values of the aluminum wedge and the specimens.

Diametral tensile strength (DTS) and setting time tests used the acrylic molds with the same size and were 6 mm and 5 mm for diameter and height, respectively. A texture analyzer machine (TA. XT plus, Stable Micro System, Godalming, UK) was used to determine the DTS values of the MTA-like cements (N = 6) at a strain rate of 6.00 mm/min. The DTS was calculated according to the following equation: DTS = 2F/πbw, where F is maximum load (N), and b and w are the diameter (mm) and the height (mm) of the cylinder, respectively. The setting times of each MTA-like cement was tested every 5 min with a Vicat needle (300 g movable rod with a needle size of 1 mm in diameter; Jin-Ching-Her Co., Ltd., Yunlin County, Taiwan). The initial setting time (N = 6) was recorded when the needle failed to create an indentation of 1 mm in depth in three separate areas, whereas the final setting time (N = 6) corresponded to that where no indentation can be observed.

Biocompatibility of MTA-like cements (12 mm in diameter and 5 mm in thickness) was evaluated by determining cell viability and attachment of human MG-63 osteoblast-like osteosarcoma cells that were purchased from the American Type Cell Culture Collection (Manassas, VA, USA). MG-63 was maintained in DMEM, 10% fetal bovine serum, 2 mM glutamine, 100 U/mL penicillin, and 100 μg/mL streptomycin at 37 °C with 5% CO_2_. The MTA extracts were incubated in 1 mL MEM medium at 37°C with 5% CO_2_ incubator for 24 h. MG-63 cells (5 × 10^4^ per well) were seeded in a 24 well plate. After incubation overnight, MG-63 cells were cultured in different MTA extracts for another 24 h and cell viability was measured using a 3-(4,5-Dimethylthiazol-2-yl)-2,5-diphenyltetrazolium bromide (MTT) assay. For cell attachment observation, MG-63 cells were seeded and incubated following the above-mentioned procedures but cultured on MTA-like cement discs. The MG-63 cells attached to cement discs were washed three times with PBS, fixed by critical point drying (CPD), gold coated, and observed by a Hitachi Tabletop TM-3000 Scanning Electron Microscope (Hitachi Ltd., Tokyo, Japan).

Statistical investigations among various MTA-like cements concerning radiopacity, diametral tensile strength, and cell viability were evaluated by Student’s paired t-test with a significance level of 0.05, 0.01, and 0.001 and performed using SPSS version 18.0 software (IBM Corporation, NY, USA).

## 3. Results and Discussion

### 3.1. Structural Evolution during High Energy Ball Milling

**Figure 1:**
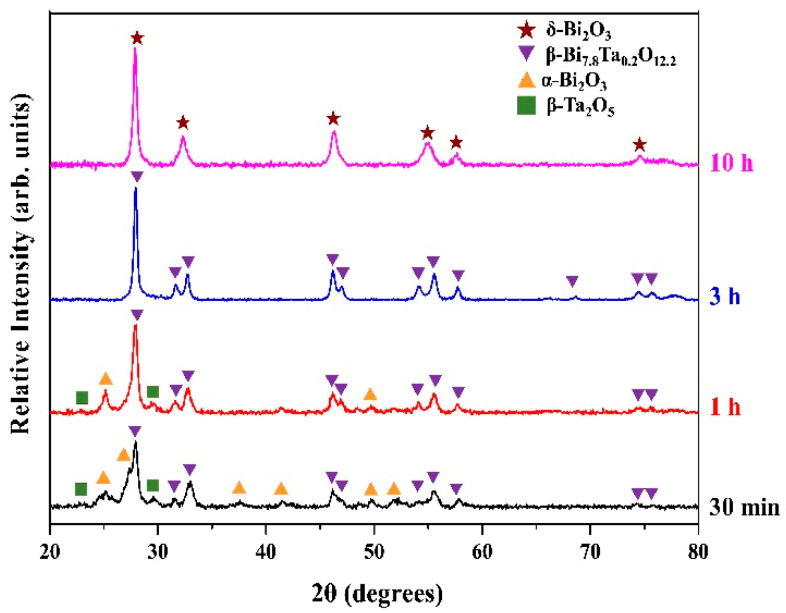
X-ray diffraction patterns of (Bi_2_O_3_)_95_(Ta_2_O_5_)_5_ powders at various milling stages.

In order to observe the phase change during the process, X-ray diffraction and scanning electron microscopy were used to examine as-milled powders at various mechanical milling stages. Figure 1 shows the XRD patterns of (Bi_2_O_3_)_95_(Ta_2_O_5_)_5_ powder at selected milling times. After 30 min of milling, it can be noted that, in addition to the diffraction peaks of the starting powders α-Bi_2_O_3_ (monoclinic structure, ICDD PDF card No. 71-0465) and β-Ta_2_O_5_ (orthorhombic, ICDD PDF card No. 89-2843), there is a new phase, β-Bi_7.8_Ta_0.2_O_12.2_ (tetragonal, ICDD PDF card No. 43-0451), formed due to the mechanochemical reaction between the starting powders α-Bi_2_O_3_ and β-Ta_2_O_5_. It is surprising to see that the amount of β-Bi_7.8_Ta_0.2_O_12.2_ was 86.4% (estimated by the Rietveld method) after 30 min of milling. By increasing milling time to 1 h, the crystalline peaks of starting powder, α-Bi_2_O_3_, were gradually replaced by the peaks of Bi_7.8_Ta_0.2_O_12.2_ phase (93.0% after 1 h) together with minor peaks of the β-Ta_2_O_5_ phase. With further increases in milling time to 3 h, a homogeneous single phase, Bi_7.8_Ta_0.2_O_12.2_ was exhibited. The preferred face-centered cubic δ phase, however, was not synthesized. Thus, the milling treatment was further extended to 10 h. At the end of prolonged milling treatment, it is interesting to note that β-Bi_7.8_Ta_0.2_O_12.2_ transformed further into face-centered cubic δ-Bi_2_O_3_ phase (ICDD PDF card No. 74-1373). It is suggested that the small amount of Ta_2_O_5_ addition and prolonged milling treatment facilitated the δ-Bi_2_O_3_ phase to stabilize at room temperature.

**Figure 2:**
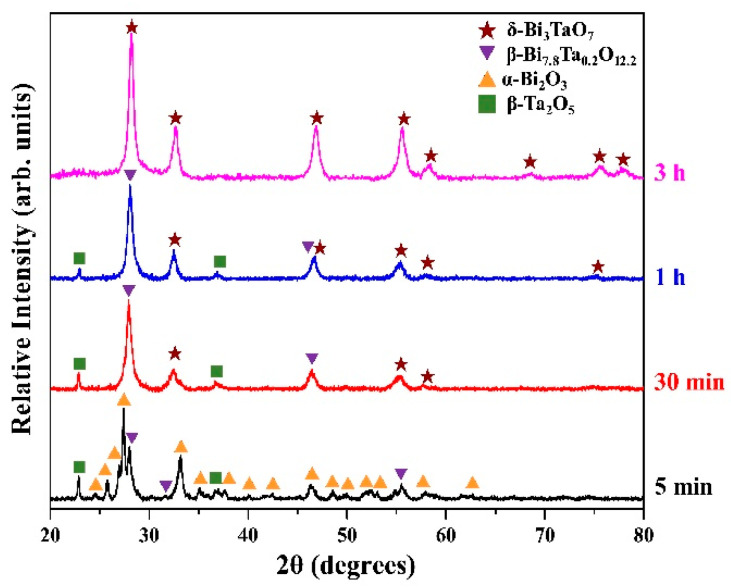
X-ray diffraction patterns of (Bi_2_O_3_)_80_(Ta_2_O_5_)_20_ powders at various milling stages.

A small amount of Ta_2_O_5_ addition can lead to the formation of high temperature metastable phases of β-Bi_7.8_Ta_0.2_O_12.2_ (tetragonal) and a δ-Bi_2_O_3_ (fcc) phase after 3 h and 10 h of high energy ball milling, respectively. When using SEM examination, however, it becomes difficult to reveal the structural evolution due to the small amount of Ta_2_O_5_ addition. In order to investigate in more detail, (Bi_2_O_3_)_80_(Ta_2_O_5_)_20_ was mechanically milled and the corresponding XRD patterns and SEM images were examined. Figure 2 shows the XRD patterns of as-milled (Bi_2_O_3_)_80_(Ta_2_O_5_)_20_ powders at various milling stages. By increasing the amount of Ta_2_O_5_ addition, it can be observed that β-Bi_7.8_Ta_0.2_O_12.2_ (46.0%) was formed after only 5 min of milling. During the short milling times (say up to 25 min), diffraction peaks of starting powders (α-Bi_2_O_3_ and β-Ta_2_O_5_) decreased and broadened continuously and were accompanied by the increase of β-Bi_7.8_Ta_0.2_O_12.2_ diffraction peak intensities. After 25 min of milling, the as-milled powder consisted of a major β-Bi_7.8_Ta_0.2_O_12.2_ phase (94.3%) and minor α-Bi_2_O_3_ (4.0%) and β-Ta_2_O_5_ (1.7%) phases. Prolonged milling enabled the reaction of Ta_2_O_5_ with the δ-Bi_2_O_3_ matrix, transformation of β-Bi_7.8_Ta_0.2_O_12.2_ phase, and resulted in the formation of another new phase, face-centered cubic δ-Bi_3_TaO_7_ (ICDD PDF card No. 44-0202). Part of the β-Bi_7.8_Ta_0.2_O_12.2_ phase transformed into δ-Bi_3_TaO_7_ phase after 30 min of milling. Compared to that of (Bi_2_O_3_)_95_(Ta_2_O_5_)_5_ shown in Figure 1, the formation of δ-Bi_2_O_3_ phase was observed after 10 h of milling. However, with a limited amount of Ta_2_O_5_ addition, only the δ-Bi_2_O_3_ phase (not the δ-Bi_3_TaO_7_ phase, they have slightly different peak locations) can be prepared. The superfluous amount of Ta_2_O_5_ in the (Bi_2_O_3_)_80_(Ta_2_O_5_)_20_ system accelerated the formation of a high temperature δ-Bi_3_TaO_7_ phase after merely 30 min of milling. As revealed by the XRD results, the milling of α-Bi_2_O_3_ and β-Ta_2_O_5_ starting powders will result in a sequence of phase transitions from β-Bi_7.8_Ta_0.2_O_12.2_, δ-Bi_2_O_3_, and the δ-Bi_3_TaO_7_ phase. Increasing the amount of tantalum oxide and milling times can speed up the formation of these high temperature metastable phases. Table 1 summarizes the crystalline phases for (Bi_2_O_3_)_95_(Ta_2_O_5_)_5_ and (Bi_2_O_3_)_80_(Ta_2_O_5_)_20_ after different milling times.

**Figure 3:**
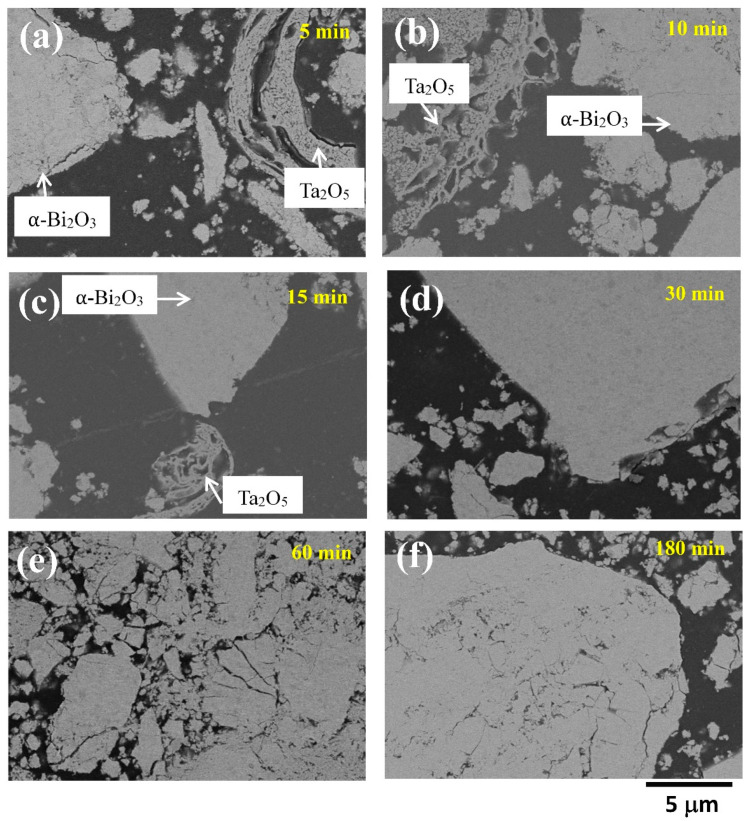
SEM images of (Bi_2_O_3_)_80_(Ta_2_O_5_)_20_ powders after (**a**) 5, (**b**) 10, (**c**) 15, (**d**) 30, (**e**) 60, and (**f**) 180 min of milling.

Mechanical milling of a mixture of α-Bi_2_O_3_ and β-Ta_2_O_5_ is expected to be different from the original mechanical alloying process, where two metallic elements undergo repetitive deformation, cold welding, and fracturing. A lamellar structure forms at early stages of milling, continuously refines, and becomes a uniform new phase at the end of the alloying process. Compared to ductile metallic elements, however, both α-Bi_2_O_3_ and β-Ta_2_O_5_ are brittle and expected to fracture and entangle with each other. Gradually, mechanochemical reaction occurs and new phases form with the aid of high impact energy input during ball milling. The microstructural evolution was examined by SEM on the cross-sectional views of as-milled powders. Figure 3 shows a series of as-milled powders after different milling times. As shown in Figure 3a for as-milled powders after 5 min of milling, one can note large particles with a relatively white color and numerous small fragments mixed with tiny white and gray particles. According to backscatter electron images and EDS mapping (Figure S1), the particles of white color were bismuth rich and should be α-Bi_2_O_3_, whereas the tiny gray particles were β-Ta_2_O_5_, as indicated by the arrows in Figure 3a. By increasing milling time to 10 and 15 min (Figure 3b,c), tinier white Bi_2_O_3_ particles mingling with gray β-Ta_2_O_5_ particles can be observed. Though β-Ta_2_O_5_ can be observed by XRD (Figure 2), it is difficult to distinguish using SEM after 30 and 60 min of milling (Figure 3d,e). This indicates that the tiny Ta_2_O_5_ particles were embedded into the bismuth-rich matrix (β-Bi_7.8_Ta_0.2_O_12.2_ or δ-Bi_3_TaO_7_ as revealed by XRD). Figure 3f exhibited uniform color distribution attributed to a single δ-Bi_3_TaO_7_ phase.

### 3.2. Performance of MTA-like Cements

**Figure 4:**
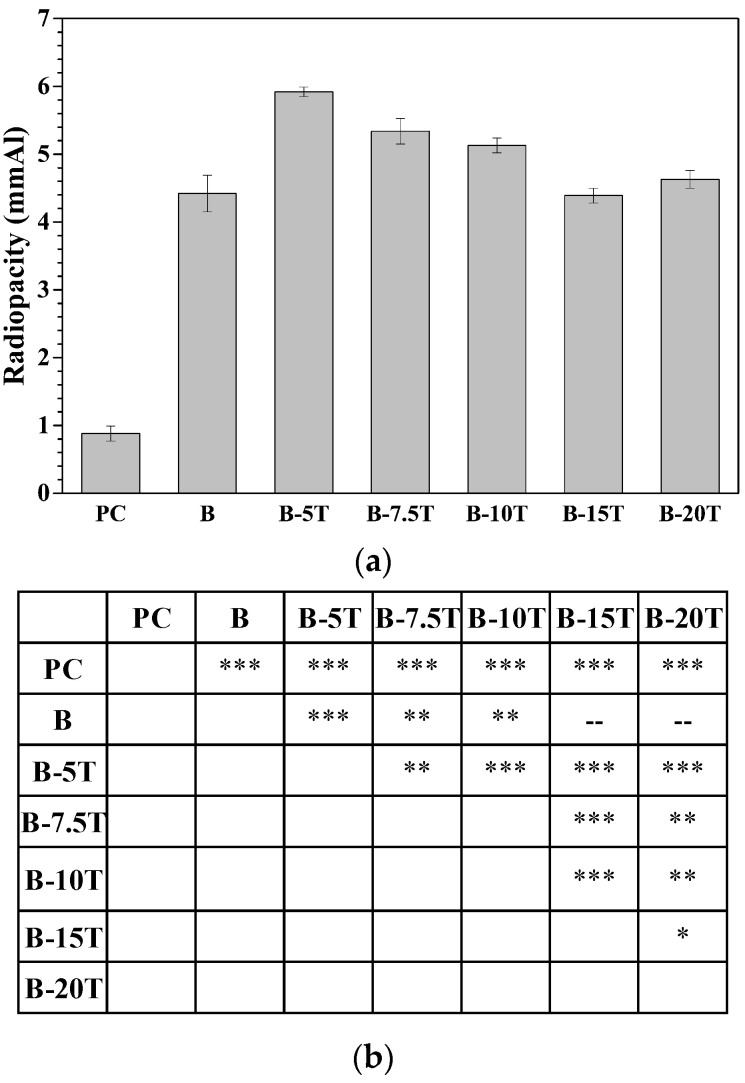
(**a**) Radiopacities of MTA-like cements prepared by using (Bi_2_O_3_)_100−x_(Ta_2_O_5_)_x_ (x = 0, 5, 7.5, 10, 15, and 20, i.e., B, B-5T, etc.) as radiopacifiers, (**b**) detailed analyses where “--” designated no difference, *, **, and *** indicated that these two sets of samples were statistically different at a 95, 99, and 99.9% confidence intervals, respectively.

Though as-milled powders prepared at various stages consisted of different phases, the radiopacity was affected mainly by the density and atomic number of MTA-like cements. Previous investigations concerning MTA-like cements prepared by as-milled (Bi_2_O_3_)_100−x_(ZrO_2_)_x_ composite powders revealed that the radiopacity was relatively high at either the early stage or the end of milling. In addition, the radiopacity decreased with increasing amounts of zirconia addition [24]. Thus, the (Bi_2_O_3_)_95_(Ta_2_O_5_)_5_ powders after 30 min and 3 h of milling were chosen to prepare MTA-like cements and the corresponding radiopacities were 5.92 ± 0.07 mmAl and 5.83 ± 0.09 mmAl, respectively. According to Table 1, where percentages of individual phases at various milling times were shown, the 30 min as-milled (Bi_2_O_3_)_95_(Ta_2_O_5_)_5_ powder consisted of β-Bi_7.8_Ta_0.2_O_12.2_ (86.4%), α-Bi_2_O_3_ (12.8%), and β-Ta_2_O_5_ (0.8%). The 3 h as-milled (Bi_2_O_3_)_95_(Ta_2_O_5_)_5_ powder exhibited a β-Bi_7.8_Ta_0.2_O_12.2_ (100%) phase. The density for α-Bi_2_O_3_, β-Ta_2_O_5_, and β-Bi_7.8_Ta_0.2_O_12.2_ is 9.37, 8.31, and 9.18 g/cm^3^, respectively. The measured radiopacity did not follow the expected rule [26]. It is suggested that, in addition to the composition, the particle size distribution at various stages may affect the solidification of MTA-like cements and the radiopacity performance. No significant differences (as-milled 30 min and 3 h), however, can be observed and showed a similar trend as that reported in the literature [24]. Thus, 30 min as-milled powder was used as the radiopacifier in MTA to further investigate the effects of tantalum pentoxide addition. Figure 4 shows the radiopacity of MTA-like cements prepared by various (Bi_2_O_3_)_100−x_(Ta_2_O_5_)_x_ (x = 0, 5, 7.5, 10, 15, and 20; coded as B, B-5T, etc.) composite powders. As shown in Figure 4a, the radiopacity of Portland cement was 0.88 ± 0.11 mmAl and increased significantly to 4.42 ± 0.27 mmAl with Bi_2_O_3_ as the radiopacifier. The (Bi_2_O_3_)_100−x_(Ta_2_O_5_)_x_ composite powder increased the radiopacities further to 5.92 ± 0.07, 5.34 ± 0.19, 5.13 ± 0.11, 4.39 ± 0.11, and 4.63 ± 0.13 mmAl, with 5, 7.5, 10, 15, and 20 wt.% Ta_2_O_5_ addition, respectively. A small amount of tantalum oxide addition (5 wt.%, i.e., B-5T) exhibited the highest radiopacity of 5.92 mmAl and decreased generally with increasing Ta_2_O_5_ addition. In order to better distinguish the statistical differences among these radiopacifiers, a more detailed analysis was shown in Figure 4b, where statistical differences at 95, 99, and 99.9% confidence intervals were presented. B-5T (the one with the highest radiopacity) was statistically different at a 99% confidence interval from B-7.5T, and statistically different at a 99.9% confidence interval with the rest of the samples. Table S1 summarizes the radiopacities and corresponding statistical analyses of MTA-like cements prepared by using (Bi_2_O_3_)_100−x_(Ta_2_O_5_)_x_ (x = 0, 5, 7.5, 10, 15, and 20, i.e., B, B-5T, etc.) as radiopacifiers.

**Figure 5:**
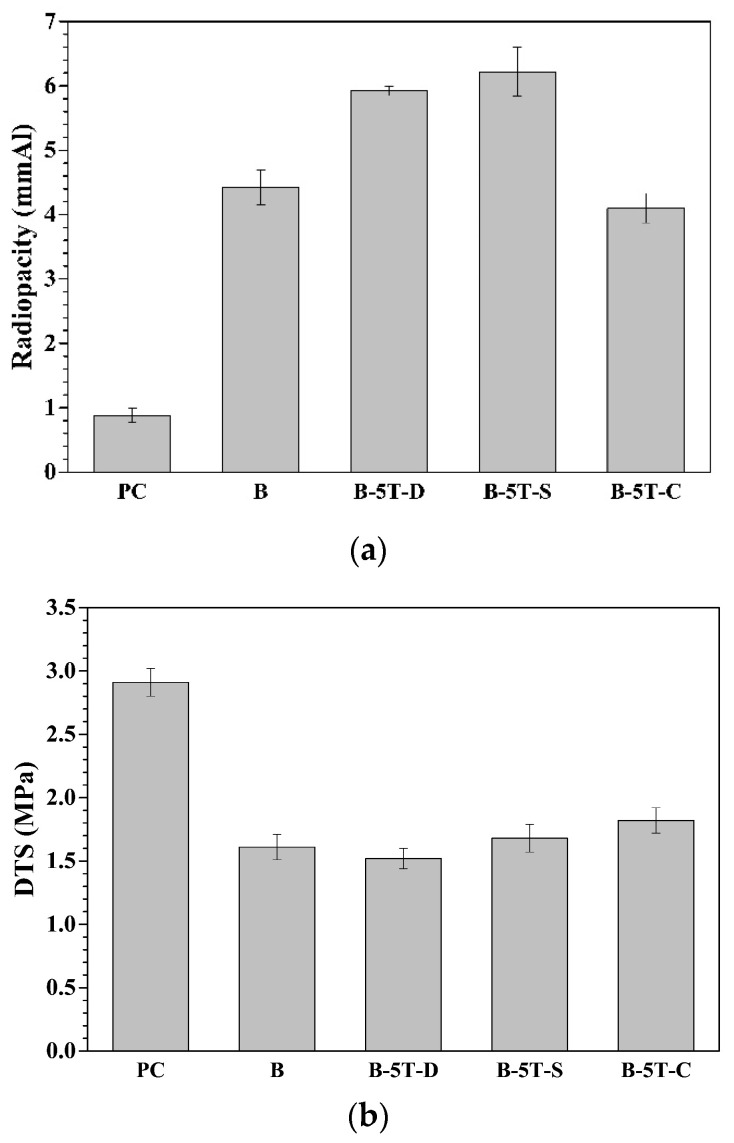

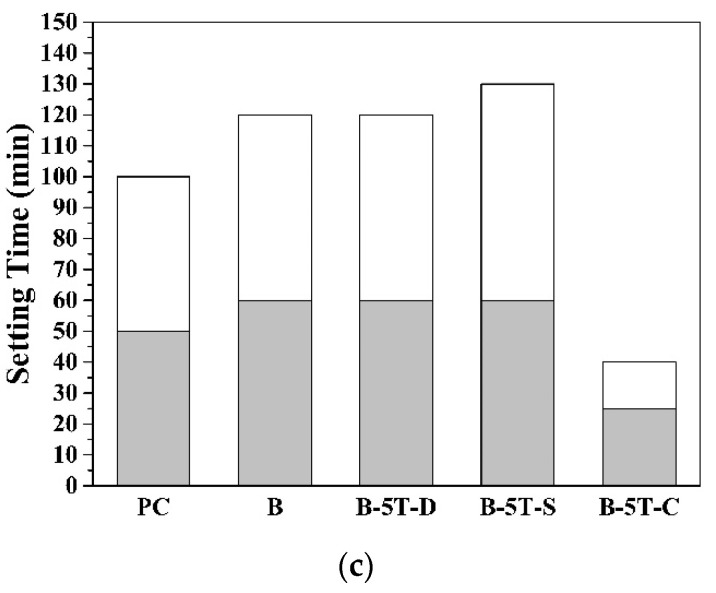
(**a**) Radiopacities, (**b**) DTS, and (**c**) setting times of MTA-like cements prepared by B-5T as a radiopacifier and solidified by various solutions where D, C, and S represent DI water, calcium chloride, and saline, respectively.

In addition to the radiopacity performance, diametral tensile strength (Figure S2) of the corresponding MTA-like cements was measured and ranged from 1.52 to 1.75 MPa without any statistical differences at a 95% confidence interval. No monotonic trend concerning DTS results as a function of Ta_2_O_5_ addition were noted. B-5T (i.e., (Bi_2_O_3_)_95_(Ta_2_O_5_)_5_, after 30 min of milling), however, is the one with the highest radiopacity and is statistically different from the other samples. Further investigations will be focused on the B-5T sample. Figure 5 shows the radiopacity, diametral tensile strength (i.e., DTS), and setting times of MTA-like cements prepared by using B-5T and solidified with various solutions. Portland cement (PC) and bismuth oxide (B) were solidified with DI water for comparison. As shown in Figure 5a, the radiopacity of B-5T-D (the one solidified using deionized water) was 5.92 ± 0.07 mmAl. It increased to 6.22 ± 0.38 mmAl using saline water (B-5T-S) but decreased to 4.10 ± 0.23 mmAl (which still meets the 3 mmAl radiopacity requirement) using 10% calcium chloride solution (B-5T-C). Diametral tensile strength, shown in Figure 5b, did not show significant differences when solidified with various solutions. The DTS was 1.52 ± 0.08, 1.68 ± 0.11, and 1.82 ± 0.10MPa for B-5T-D, B-5T-S, and B-5T-C, respectively. The DTS was similar to that of Bi_2_O_3_ solidified with DI water (sample B, 1.61 ± 0.10 MPa), but smaller than that of PC (2.91 ± 0.11 MPa), whereas the setting times were similar for samples PC, B, B-5T-D, and B-5T-S. Accelerated solidification, however, was noted for B-5T-C (B-5T solidified with 10% calcium chloride solution) where the initial and final setting times of 25 and 40 min, respectively. The final setting time (40 min) was even shorter than the initial setting times (50 min for PC, and 60 min for B, B-5T-D, and B-5T-S) for the other samples. Table 2 summarizes the radiopacities, diametral tensile strengths (DTS), and setting times of MTA-like cements prepared by B-5T and solidified by various solutions.

**Figure 6:**
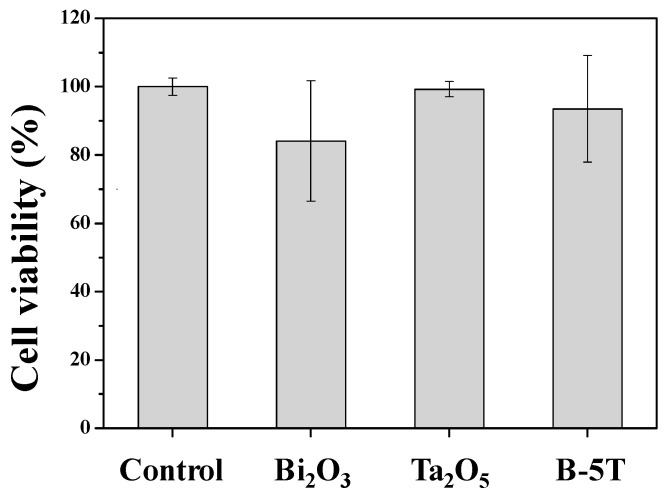
Cell viability of MTA-like cements prepared by various radiopacifiers where B-5T represents (Bi_2_O_3_)_95_(Ta_2_O_5_)_5_ powder.

**Figure 7:**
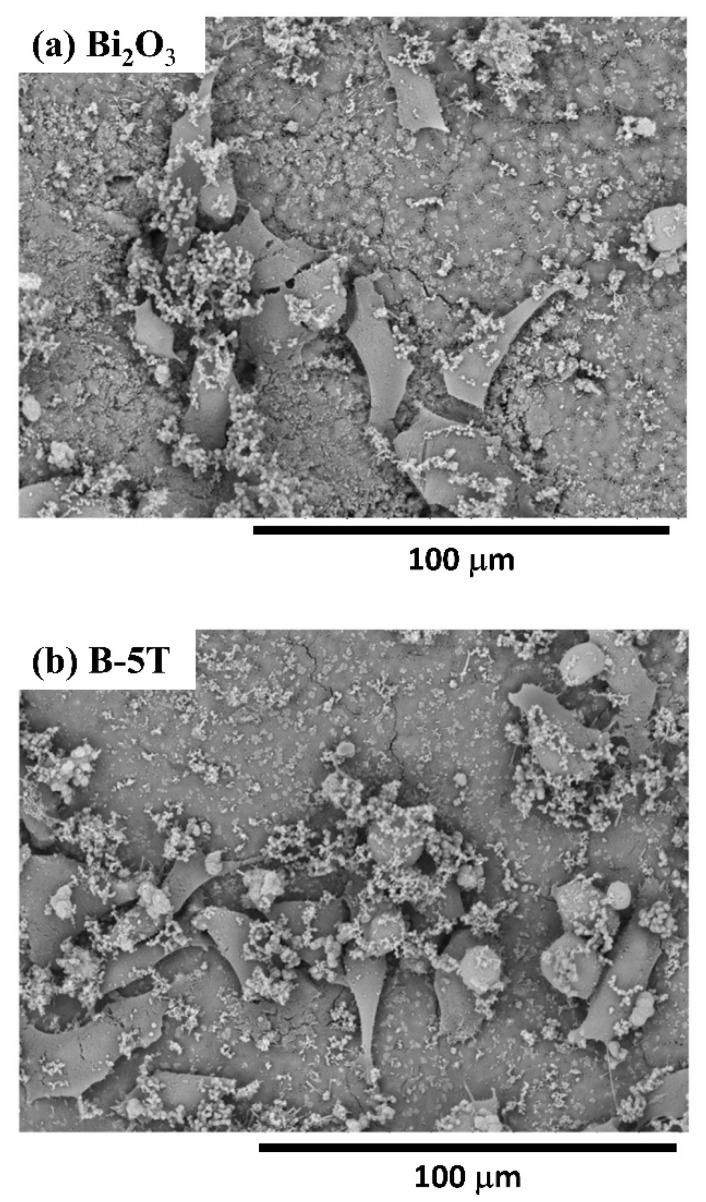
Scanning electron microscopy images of MG-63 cells attached on (**a**) Bi_2_O_3_ and (**b**) B-5T MTAs.

Radiopacities, diametral tensile strength, and setting times of MTA-like cements using various Ta_2_O_5_-added Bi_2_O_3_ composite powders have been investigated. The biocompatibility in selected cement extracts was further examined. As shown in Figure 6, all the cement extracts revealed no significant cytotoxicity effects in MG-63 osteoblast-like cells, which indicated all MTA-like cements had good biocompatibility. Though Bi_2_O_3_–cement extract exhibited MG-63 cell viability of 84 ± 18%, Ta_2_O_5_ possessed the highest cell viability of 99 ± 2%, and that of B-5T was in between these (94 ± 16%). Interestingly, MTA-contained Bi_2_O_3_ revealed a higher standard deviation than Ta_2_O_5_. Bi_2_O_3_ is a reactive oxygen species (ROS) generator and causes toxic effects in human cancer cells [27,28]. For MG63 cell viability, the present results exhibited no cytotoxicity but a relatively large standard deviation and showed a similar trend as that reported by Attik et al. [29]. In contrast to Bi_2_O_3_, Ta_2_O_5_ does not cause cytotoxicity in human skin fibroblast cells [30]. Ta_2_O_5_ and B-5T may harbor less toxicity and better biocompatibility than Bi_2_O_3_ for MTA-like cements. Furthermore, we examined the MG-63 cell attaching ability on MTA-like cements prepared by using Bi_2_O_3_ and B-5T (Figure 7a,b, respectively). Compared to its Bi_2_O_3_ counterpart (Figure 7a), MG-63 cells on B-5T MTA (Figure 7b) showed fibroblast-like morphology and had more cell–cell connections and adhesion attachments. In general, B-5T was superior to its pristine counterpart (Bi_2_O_3_) in radiopacity, diametral tensile strength, setting times, and biocompatibility. Further investigations concerning tooth discoloration and in vivo animal testing will be performed before clinical applications.

## 4. Conclusions

Mechanical milling of (Bi_2_O_3_)_95_(Ta_2_O_5_)_5_ or (Bi_2_O_3_)_80_(Ta_2_O_5_)_20_ powder mixtures will induce subsequent formation of high temperature metastable β-Bi_7.8_Ta_0.2_O_12.2_, δ-Bi_2_O_3_, and δ-Bi_3_TaO_7_ phases. The more the amount of tantalum pentoxide added, the shorter the required milling time for the formation of these phases. As-milled powders were used as the radiopacifiers for MTA-like cements. The radiopacity was not affected by the milling time but by the amount of tantalum pentoxide addition. MTA-like cement with a small amount of tantalum oxide addition (5 wt.%; i.e., B-5T) exhibited the highest radiopacity of 5.92 mmAl and generally decreased with increasing Ta_2_O_5_ addition (4.63 mmAl for 20 wt.%). In general, Ta_2_O_5_-added Bi_2_O_3_ composite powder exhibited better radiopacity performance than Portland cement (0.88 mmAl) and its Bi_2_O_3_ counterpart (4.42 mmAl). In addition to radiopacity performance, the Bi_2_O_3_ counterpart exhibited relatively long setting times (60 and 120 min for initial and final setting times, respectively) and an MG-63 cell viability of 84%. B-5T solidified with 10% calcium chloride solution can further accelerate the solidification; the initial and final setting times were 25 and 40 min, respectively. The biocompatibility of B-5T was also confirmed by an MG-63 cell viability of 94% and good attachment. As compared to pristine Bi_2_O_3_, B-5T MTA-like cement exhibited superior performance in radiopacity, diametral tensile strength, setting times, and biocompatibility.

## Figures and Tables

**Table 1:** Crystalline phases of (Bi_2_O_3_)_95_(Ta_2_O_5_)_5_ and (Bi_2_O_3_)_95_(Ta_2_O_5_)_5_ at various stages of ball milling.

Composition	Milling Time	Crystalline Phases *
(Bi_2_O_3_)_95_(Ta_2_O_5_)_5_	30 min	β-Bi_7.8_Ta_0.2_O_12.2_ (86.4%) + α-Bi_2_O_3_ (12.8%) + β-Ta_2_O_5_ (0.8%)
	1 h	β-Bi_7.8_Ta_0.2_O_12.2_ (93.0%) + α-Bi_2_O_3_ (6.6%) + β-Ta_2_O_5_ (0.4%)
	3 h	β-Bi_7.8_Ta_0.2_O_12.2_ (100%)
	10 h	δ-Bi_2_O_3_ (100%)
(Bi_2_O_3_)_80_(Ta_2_O_5_)_20_	5 min	α-Bi_2_O_3_ (47.3%) + β-Ta_2_O_5_ (6.7%) + β-Bi_7.8_Ta_0.2_O_12.2_ (46.0%)
	10 min	α-Bi_2_O_3_ (28.6%) + β-Ta_2_O_5_ (6.2%) + β-Bi_7.8_Ta_0.2_O_12.2_ (65.2%)
	15 min	α-Bi_2_O_3_ (12.3%) + β-Ta_2_O_5_ (3.1%) + β-Bi_7.8_Ta_0.2_O_12.2_ (88.1%)
	20 min	α-Bi_2_O_3_ (7.1%) + β-Ta_2_O_5_ (1.7%) + β-Bi_7.8_Ta_0.2_O_12.2_ (91.1%)
	25 min	α-Bi_2_O_3_ (4.0%) + β-Ta_2_O_5_ (1.7%) + β-Bi_7.8_Ta_0.2_O_12.2_ (94.3%)
	30 min	β-Ta_2_O_5_ (0.9%) + β-Bi_7.8_Ta_0.2_O_12.2_ (73.2%) + δ-Bi_3_TaO_7_ (25.9%)
	1 h	β-Ta_2_O_5_ (0.6%) + β-Bi_7.8_Ta_0.2_O_12.2_ (64.0%) + δ-Bi_3_TaO_7_ (35.4%)
	3 h	δ-Bi_3_TaO_7_ (100%)

* The percentage of the individual phase is given in the bracket.

**Table 2:** Radiopacities, diametral tensile strength (DTS), and setting times of MTA-like cements prepared by B-5T as a radiopacifier and solidified by various solutions where D, C, and S represent DI water, calcium chloride, and saline, respectively.

	Properties	Radiopacities (mmAl)	DTS (MPa)	Initial Setting Time (min)	Final Setting Time (min)
Sample					
PC		0.88 ± 0.11	2.91 ± 0.11	50	100
B		4.42 ± 0.27	1.61 ± 0.10	60	120
B-5T-D		5.92 ± 0.07	1.52 ± 0.08	60	120
B-5T-S		6.22 ± 0.38	1.68 ± 0.11	60	130
B-5T-C		4.10 ± 0.23	1.82 ± 0.10	25	40

## Data Availability

Not applicable. But it may be obtained from the corresponding authors upon request.

## Supplementary Materials

The following are available online at https://www.mdpi.com/article/10.3390/ma14237447/s1. Table S1. Radiopacities (in mmAl) of MTA-like cements prepared by using (Bi_2_O_3_)_100−x_(Ta_2_O_5_)_x_ (x = 0, 5, 7.5, 10, 15, and 20, i.e., B, B-5T, etc.) as radiopacifiers. Statistical analyses were presented by mean, standard deviation, and 95, 99, and 99.9% confidence intervals.; Figure S1. (a) SEM and (b) EDS mapping of as-milled BiTaO_x_ powder where region 1 (relative white, red curve) was Bi-rich and region 2 was Ta-rich (the blue one).; Figure S2. Diametral tensile strength of MTA-like cements prepared by using (Bi_2_O_3_)_100−x_(Ta_2_O_5_)_x_ (x = 0, 5, 7.5, 10, 15, and 20, i.e., B, B-5T, etc.) as radiopacifiers.
