# Supplementary material for: Effect of Tantalum Pentoxide Addition on the Radiopacity Performance of Bi2O3/Ta2O5 Composite Powders Prepared by Mechanical Milling
Source: Materials (Basel). 2021 Dec 4;14(23):7447. doi: 10.3390/ma14237447 (PMC8659089; doi:10.3390/ma14237447)
Supplement: Supplementary file 1 [file materials-14-07447-s001.zip › materials-1443963-supplementary.pdf]

Table S1. Radiopacities (in mmAl) of MTA-like cements prepared by using $(Bi_2O_3)_{100-x}(Ta_2O_5)_x$ (x=0, 5, 7.5, 10, 15, and 20, i.e., B, B-5T, etc.) as radiopacifiers. Statistical analyses were presented by mean, standard deviation, and 95, 99, and 99.9% confidence intervals.

Statistics / Sample	Mean	S.D.	Lower 95% C.I.	Upper 95% C.I.	Lower 99% C.I.	Upper 99% C.I.	Lower 99.9% C.I.	Upper 99.9% C.I.
PC	0.88	0.11	0.76	1.00	0.70	1.06	0.57	1.19
B	4.42	0.27	4.14	4.70	3.98	4.86	3.66	5.18
B-5T	5.92	0.07	5.85	5.99	5.80	6.04	5.72	6.12
B-7.5T	5.34	0.19	5.14	5.54	5.03	5.65	4.81	5.87
B-10T	5.13	0.11	5.01	5.25	4.95	5.31	4.82	5.44
B-15T	4.39	0.11	4.27	4.51	4.21	4.57	4.08	4.70
B-20T	4.63	0.13	4.49	4.77	4.42	4.84	4.27	4.99

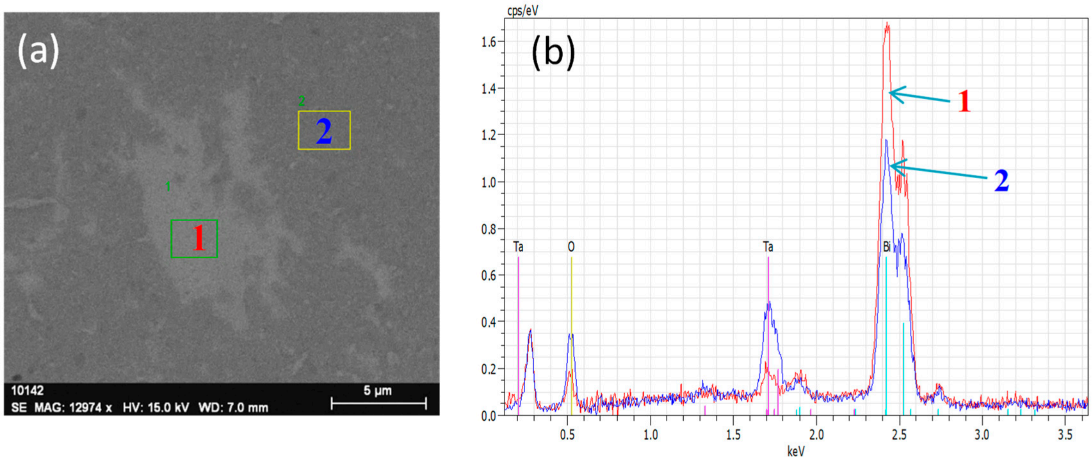

Figure S1. (a) SEM and (b) EDS mapping of as-milled BiTaOx powder where region 1 (relative white, red curve) was Bi-rich and region 2 was Ta-rich (the blue one)

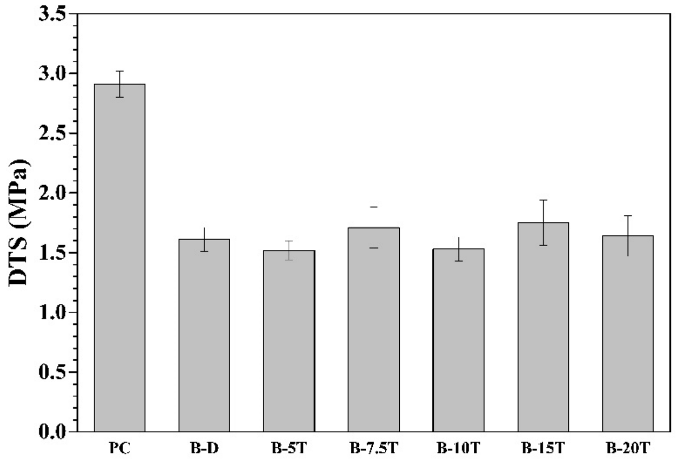

Figure S2. Diametral tensile strength of MTA-like cements prepared by using $(Bi_2O_3)_{100-x}(Ta_2O_5)_x$ (x=0, 5, 7.5, 10, 15, and 20, i.e., B, B-5T, etc.) as radiopacifiers.
